# Group B *Streptococcus* vaginal colonisation throughout pregnancy is associated with decreased *Lactobacillus crispatus* and increased *Lactobacillus iners* abundance in the vaginal microbial community

**DOI:** 10.3389/fcimb.2024.1435745

**Published:** 2024-09-30

**Authors:** Toby I. Maidment, Elise S. Pelzer, Danielle J. Borg, Eddie Cheung, Jake Begun, Marloes Dekker Nitert, Kym M. Rae, Vicki L. Clifton, Alison J. Carey

**Affiliations:** ^1^ Centre for Immunology & Infection Control, School of Biomedical Sciences, Queensland University of Technology, Brisbane, QLD, Australia; ^2^ Mater Research Institute, University of Queensland, Brisbane, QLD, Australia; ^3^ Faculty of Medicine, The University of Queensland, Brisbane, QLD, Australia; ^4^ Mater Young Adult Health Centre, Mater Misericordiae Ltd, Brisbane, QLD, Australia; ^5^ Mater Clinical School and Princess Alexandra Clinical School, School of Medicine, University of Queensland, St. Lucia, QLD, Australia; ^6^ School of Chemistry and Molecular Biosciences, The University of Queensland, St. Lucia, QLD, Australia

**Keywords:** Group B *Streptococcus*, vaginal microbiome, pregnancy, *Lactobacillus* sp., *Lactobacillus iners*

## Abstract

Group B *Streptococcus* (GBS) asymptomatically colonises the vagina of up to 40% of pregnant women and can transmit to neonates during birth, causing neonatal pneumonia, sepsis, meningitis, and significant mortality. Vaginal GBS colonisation can be attributed to a range of host and bacterial factors, which may include the composition of the vaginal microbial community. There are few studies that have examined the vaginal community composition in relation to GBS colonisation throughout pregnancy. Here, we performed 16S rRNA sequencing (V3-V4) on vaginal swabs from women at 24- and 36-weeks’ gestation, who were GBS culture-negative or GBS culture-positive at either 24 weeks or 36 weeks’ gestation or at both timepoints. Vaginal swabs from 93 women were analysed; 46 women were culture-negative, 11 women GBS culture-positive at 24 weeks only, 21 women GBS culture-positive at 36 weeks only and 15 women GBS culture-positive at both timepoints on *Brilliance* GBS agar. V3-V4 16S rRNA gene amplicon sequencing demonstrated that in women that were GBS culture-positive at 36 weeks gestation only, *G. vaginalis* was significantly more abundant at 24-weeks’ gestation despite a lack of significant changes in community richness between the 24- and 36-week samples. The vaginal microbial communities of women persistently colonised with GBS, had a significantly higher abundance of *Lactobacillus iners*, compared to other groups where *L. crispatus, L. gasseri* or *L. jensenii* were dominant. We have characterised the vaginal microbial community composition during pregnancy in relation to GBS colonisation status, in a longitudinal study for the first time. The most interesting finding was that in women that were persistently colonised with GBS throughout pregnancy, there was a significant increase in *L. iners* and significant reduction in *L. crispatus* abundance. Given the lack of detail of the role that the vaginal microbial community plays in GBS colonisation in the literature, it is imperative that the relationship between *L. iners* and GBS in this unique environmental niche is further investigated.

## Background

*Streptococcus agalactiae* (Group B *Streptococcus*; herein GBS) is a significant cause of global neonatal and perinatal morbidity and mortality (Brokaw et al., 2021). GBS is a gastrointestinal commensal in adults, and up to 40% of pregnant women demonstrate asymptomatic vaginal GBS colonisation (Brokaw et al., 2021). Vaginal colonisation during pregnancy can lead to life-threatening invasive GBS infection of neonates through vertical transmission during delivery (Brokaw et al., 2021; Chaguza et al., 2022). Early onset disease (EOD) due to invasive GBS infection (< 7 days post-partum) can cause severe pneumonia and meningitis. GBS is the leading cause of neonatal sepsis, accounting for approximately 35% of global neonatal mortality cases (Sweeney et al., 2020; Alotaibi et al., 2023). In addition to EOD, ascending GBS infection prior to delivery is associated with an increased risk of preterm birth, stillbirth, premature rupture of membranes and fetal organ damage (Bianchi-Jassir et al., 2017; Brokaw et al., 2021; Mei and Silverman, 2023).

GBS colonisation of the vagina can be attributed to a range of host and bacterial factors. These include biological factors such as a history of ruptured membranes, age, diet, ethnicity, and gastrointestinal colonisation, as well as socioeconomic factors such as hygiene practices, occupation, illiteracy, and sexual activity (Stapleton et al., 2005; Le Doare and Heath, 2013; Capan-Melser et al., 2015; Brokaw et al., 2021). It is important to consider that GBS colonisation occurs within a distinct and typically hostile microbial ecosystem, which likely plays a determining role in facilitating or inhibiting persistent colonisation during pregnancy. Persistent GBS colonisation of the vagina occurs through the expression of several bacterial factors such as adhesins (Baron et al., 2004; Sheen et al., 2011; Jiang and Wessels, 2014), biofilm formation (D’Urzo et al., 2014), immune evasion (Jones et al., 2003; Carey et al., 2014; Kolar et al., 2015), and competitive antimicrobial defences (Gori et al., 2020). However, women can also be transiently colonised by GBS throughout pregnancy (Hansen et al., 2004) and the effects this has on pregnancy outcomes are unknown.

The vaginal microbial community exhibits hormone-dependent changes across the menstrual cycle and during pregnancy a loss of community diversity occurs, decreasing in species richness as pregnancy progresses (Ravel et al., 2011; Romero et al., 2014; Pace et al., 2021; Romero et al., 2023). A healthy vaginal microbial community is dominated by a small number of vaginal lactobacillus species (*L. crispatus, L. gasseri, L. iners, and L. jensenii*) (Ravel et al., 2011), representing community state types I, II, III and V respectively These lactobacilli populations maintain a low-pH environment (pH ~4.5) through production of lactic acid, which offers protection against colonising pathogens and is thereby an important facet of reproductive health (Ravel et al., 2011; O’Hanlon et al., 2013). Conversely, increased species diversity in the vaginal microbial community and/or dominance of other bacteria such as *Gardnerella* spp., *Prevotella* spp., *Bifidobacterium* spp., or *Atopobium* spp., can negatively alter the vaginal environment, causing vaginal dysbiosis (Ravel et al., 2011; Coudray and Madhivanan, 2020). Vaginal dysbiosis is associated with adverse maternal and neonatal sequelae; however, causality is difficult to assign to specific microorganisms (Brooks et al., 2017). GBS represents an opportunistic pathogen, present in the vaginal community of a significant number of women throughout pregnancy but is implicated in adverse outcomes only when invasion occurs (Brokaw et al., 2021). To date, characterisation of the vaginal microbial community in health and disease has primarily focussed on taxonomic classification of lactobacilli to species level; however, changes in the vaginal microbial community in the context of lactobacilli alone do not explain GBS colonisation and adverse pregnancy outcomes. Increased taxonomic diversity, *Gardnerella vaginalis* abundance, and loss of *Lactobacillus* dominance appear as possible drivers and regulators of GBS colonisation and invasion risk during pregnancy (Sroka-Oleksiak et al., 2020). In healthy, non-pregnant women GBS is not considered a pathogen, and in pregnant women GBS is only considered significant if invasion occurs. Thus, GBS has likely been disregarded in studies examining vaginal microbial communities. Indeed, the less than 30% of studies in this field reporting on GBS, highlight that this opportunistic pathogen is under-reported (Brooks et al., 2015; Lim et al., 2021).

Several cultivation-dependent investigations of the vaginal microbial community have previously identified compositional features associated with GBS colonisation similar to those in bacterial vaginosis (BV) (Bayo et al., 2002; Ronnqvist et al., 2006; Brzychczy-Wеloch et al., 2014; Tano et al., 2021). These include reductions in lactobacilli and increased populations of several BV-associated taxa such as *Gardnerella vaginalis*, *Prevotella* spp., and *Atopobium* spp., in addition to frequent co-colonisation with the opportunistic yeast *Candida albicans* (Bayo et al., 2002; Ronnqvist et al., 2006; Brzychczy-Wеloch et al., 2014; Pidwill et al., 2018; Tano et al., 2021). Using cultivation-independent analysis of the vaginal microbiota of 44 Egyptian women during the third trimester of pregnancy (Shabayek et al., 2022), Shabayek et al. (2022) similarly found GBS positive women to have significantly increased community diversity and lower abundances of *Lactobacillus* spp., compared to GBS negative women (Shabayek et al., 2022). Despite these findings however, our understanding of how GBS impacts the vaginal microbial community remains extremely limited. A considerable knowledge gap remains regarding community-wide changes in the vaginal microbial community throughout pregnancy that are associated with transient versus persistent GBS colonisation.

In this study, we used 16S rRNA gene amplicon sequencing to characterise the vaginal microbial communities of GBS culture-positive and -negative women at both 24- and 36-weeks’ gestation. We defined community-wide patterns in the vaginal microbiota associated with transient (culture-positive at one time point only) and persistent GBS carriage (culture-positive at both timepoints) during pregnancy, to better understand risk factors associated with GBS colonisation during pregnancy.

## Methods

### Participant enrolment

Participants, who freely gave informed consent, were recruited as part of the Queensland Family Cohort (QFC) prospective, longitudinal, and observational pilot study. The details of this study are published (Borg et al., 2021) and has full ethical approval by Mater Misericordiae Research Ethics committee (HREC/16/MHS/113).

### Participant data collection

At the 24- and 36- weeks’ gestation appointments the participants completed surveys on medication and lifestyle substance use. Medication use during labour and delivery, and delivery details including complications, as well as neonatal outcomes were also recorded and available. Participants were excluded from this study if they received antibiotics in the two weeks prior to vaginal swab collection, if they had diabetes (or developed gestational diabetes), or indicated they were taking probiotics. These time points represent sampling from the second and third trimester of gestation and occur prior to full term gestation (<37 weeks), where GBS may contribute to premature birth.

### Sample collection

Upon enrolment participants were given detailed instructions on how to self-collect the vaginal swab and provided with the E-swab (Copan, CA, USA). Briefly, participants were instructed to insert the swab 3-5 centimetres into the vagina and while rolling the swab, wipe the vaginal wall in 3 full circles, ensuring the swab was kept in the vagina for a minimum of 20 seconds. Participants were instructed to not use vaginal douches, feminine sprays, genital wipes, vaginal medications/suppositories, or have sexual intercourse 48 hours prior to swab collection. Swabs were collected at the participant’s 24- and 36-week gestation maternal appointments and stored at -80°C until use. Placentae were observed at the time of birth and classified as normal/abnormal by the collector at the time.

### GBS screening & characterisation

All swabs were thawed and cultured on *Brilliance* GBS agar plates (ThermoFisher, Seventeen Mile Rocks QLD, Australia) using the 16-streak dilution technique as per Australian diagnostic standards. Plates were incubated at 37°C, atmospheric conditions (O_2_) for 24-48 hours and the growth of GBS was recorded as per the manufacturer’s instructions. Well isolated, individual GBS colonies were sub-cultured onto 5% Horse blood agar containing Colistin and Nalidixic Acid (CNA; ThermoFisher). Colony characteristics were recorded and well isolated colonies from the CNA were used to determine the serotype of the GBS using the ImmuLex Streptococcus-B kit (SSI Diagnostica, Denmark) as per manufacturer’s instructions.

### Vaginal swab DNA extraction

Based on the GBS culture results participants were grouped into either GBS culture-negative, GBS culture-positive at 24 weeks or 36 weeks’ gestation only, or GBS culture-positive at both 24- and 36-weeks’ gestation. Vaginal swabs were thawed on ice, vortexed vigorously to suspend the bacterial cells into the liquid Amies solution and 500 μL of the liquid Amies was transferred to a sterile 2 mL tube on ice. For cell lysis, 50 μL of lysozyme (10 mg/ml stock; Sigma-Aldrich, NSW, Australia), 6 μL of mutanolysin (25,000 U/ml stock; Sigma-Aldrich), 3 μL of lysostaphin (4,000 U/ml in sodium acetate stock; Sigma-Aldrich), and 41 μL of TE50 buffer (10 mM Tris-HCl, 50 mM EDTA [pH 8.0]) were added to each sample. Samples were incubated at 37°C for 1 hr, then 10 μL of proteinase K (20 mg/mL stock; Qiagen, VIC, Australia), 100 μL of 10% sodium dodecyl sulfate, and 20 μL of RNase A (20 mg/ml stock; ThermoFisher) to each sample. Samples were incubated for 1 hr at 55°C. Following enzymatic lysis, samples were mechanically disrupted and homogenized using a Biospec Mini-BeadBeater 16 (Biospec, Oklahoma, USA). DNA was extracted using the QIAamp DNA mini kit (Qiagen) as per manufacturer’s instructions, omitting the recommended lysis step, as enzymatic and physical lysis was completed above. DNA was eluted using the molecular grade water provided with the Qiagen kit, pre-warmed to 56°C.

### 16S rRNA amplicon sequencing

Library preparation and 16S rRNA gene amplicon sequencing was performed at the Australian Genome Research Facility (Melbourne, Victoria, Australia) using the Illumina MiSeq platform with 2x300bp chemistry. Sequencing was performed using a modified 319F (5’-CCTACGGGAGGCAGCAGT-3’) primer and 806R (5’-GGACTACHVGGGTWTCTAAT-3’) primer targeting the V3-V4 hypervariable region, which were selected due to use in prior investigations of the vaginal microbial communities (Graspeuntner et al., 2018; Van Der Pol et al., 2019; Romero et al., 2023).

### Bacterial community profiling & statistics

Demultiplexed, 300-bp paired-end reads were imported into Quantitative Insights into Microbial Ecology-2 (QIIME2; v2021.11) (Bolyen et al., 2019), after which adapter sequences were removed using *Cutadap*t (Kechin et al., 2017) and reads quality checked using Q2-*Demux.* Denoising and amplicon sequence variant (ASV) assignment were performed on quality-filtered reads using the *Deblur* ‘denoise-16S’ tool (Amir et al., 2017). Denoised representative sequence outputs were then assigned taxonomy using a region-specific V3-V4 taxonomic classifier with the *Classify-sklearn* tool (Bokulich et al., 2018), which was built using *q2-rescript* (Robeson et al., 2021) with the SILVA database (SSUr138, NR_99; https://www.arb-silva.de/), and trained using the *fit-classifier-naïve-bayes* tool prior to use. Following this, the feature table was filtered to remove rare taxa (< 2 samples), taxa unassigned past the domain level, chloroplast sequences, and mitochondrial sequences. As contamination is commonplace in 16S rRNA gene amplicon data, taxonomic data was screened for putative contaminants using *Decontam* (Davis et al., 2018), with suspected contaminant ASVs identified and filtered from feature data based on their prevalence in negative controls (threshold = 0.5). Assignment of vaginal community state types (CSTs) to individual samples was performed manually based on the relative abundance of important taxa outlined by France et al. (2020), including sub-types for CSTs I, III, and IV (France et al., 2020). Subsequent visualisation of taxonomic and CST data was performed using QIIME2 (v2021.11) in addition to the R packages *ggplot2* (v3.4.1)*, Microbiome* (v1.16.0), and *Phyloseq* (v1.38.0) (Lahti and Shetty, [[NoYear]]; Wickham et al., [[NoYear]]; McMurdie and Holmes, 2013).

Rooted and unrooted phylogenetic trees used for subsequent analysis were then produced using the *align-to-tree-mafft-fasttree* QIIME2 command, after which generic alpha and beta diversity calculations were generated using the *q2-diversity core-metrics phylogenetic* tool from a feature table rarefied to 17,000 reads per sample. Statistically significant differences in Shannon entropy between sample groups were identified *via* Kruskal-Wallis test, using the *q2-diversity alpha-group-significance* tool. Statistical differences in community structure were calculated using Analysis of Similarity (ANOSIM) testing on unweighted Unifrac distances with 4000 permutations. Differential abundance testing was performed with DeSeq2 (Love et al., 2014) via *Phyloseq* (McMurdie and Holmes, 2013), using the geometric means of CLR-transformed count data. The Wald test with Benjamini-Hochberg multiple test corrections was used to identify taxa which significantly differed in abundance between groups, with an FDR-adjusted P-value <0.05 deemed statistically significant.

### Negative and positive sequencing controls

To ensure the validity of the low biomass vaginal samples, several extraction controls were included. This included a negative swab control where the swab was removed from the sterile transport tube exposed to the air where extractions were to be performed and sample lysis and DNA extraction was performed as described above. There was a DNA extraction kit control, where the water used to elute the DNA was used as a ‘sample’ and underwent DNA extraction. These controls were completed to account for possible kit and environmental contamination. A Gram-positive and Gram-negative positive control were also included and consisted of a combination of *Enterococcus faecalis* (ATCC29212) and *Escherichia coli* (ATCC8739), respectively (Maidment et al., 2023).

## Results

### Participant demographics

Women were recruited to the pilot QFC study at Mater Hospital and at their 24- and 36-weeks’ gestation appointment, completed surveys and provided a self-collected vaginal swab (n = 209). Vaginal swabs from 93 women were analysed and based on the culture of vaginal swabs were grouped as follows: GBS culture-negative at both 24- and 36-weeks’ gestation (GBS NEG; n = 46 women), GBS culture-positive at 24 weeks’ only (GBS 24 wk; n = 11 women), GBS culture-positive at 36 weeks’ only (GBS 36 wk; n = 21 women) and GBS culture-positive at both 24- and 36-weeks’ gestation (GBS 24/36 wk; n = 15 women). There was no difference in the maternal age or gestational age at birth between the groups and women were primarily of Caucasian decent (**Table 1**). Interestingly in those women that were GBS culture-positive at either or both collection timepoints, there was a 1.5 – 2-fold increase in observed placental abnormality, albeit not significant (**Table 1**).

**Table 1:** QFC demographics.

Group	Maternal age at conception (median) [P value]	Baby gestational age range (median) [P value]	Ethnicity (%)	Placenta abnormality (%)
GBS NEG (46 women)	21 – 40 (30)	36.5 – 41.2 (39.35)	Caucasian (67.4), Latin American (8.7), North-East Asian, African, Aboriginal, Eastern European (2.2 each), Southern-Central Asian (4.3), South-East Asian (6.5), Undefined (4.3)	23.9% (n/a 15.2%)
GBS POS 24 wk (11 women)	20 – 42 (30) [P = 0.735]	36.2 – 40.5 (39.2) [P = 0.599]	Caucasian (81.8), South-East Asian (18.2)	45.5% (n/a 18.2%) [P = 0.666]
GBS POS 36 wk (21 women)	24 – 44 (32) [P = 0.522]	36.1 – 40.6 (39.1) [P = 0.929]	Caucasian (76.2), Southern-Central Asian (4.8), Latin American, North-East Asian (9.5 each)	38.1% (n/a 19%) [P = 0.819]
GBS POS 24 + 36 wk (15 women)	26 – 38 (34) [P = 0.224]	38.1 – 40.5 (39.1) [P = 0.866]	Caucasian (66.7), North-East Asian (6.7), Latin American (13.3), Undefined (13.3)	46.7% (n/a 6%) [P = 0.524]

GBS NEG, Culture negative for GBS at both timepoints; GBS POS, Culture positive for GBS at indicated time of gestation. Placenta abnormality n/a: placenta was not able to be examined to determine if any abnormalities were present. One way ANOVA’s with Tukey’s multiple comparison post-test was performed on maternal age, baby gestational age and placenta abnormality to compare differences between GBS positive groups and the GBS negative group (control group), with P < 0.05 set for significance.

### GBS colonisation during pregnancy is associated with a reduced vaginal *L. crispatus* abundance

16s rRNA gene amplicon sequencing of the V3-V4 variable region was performed on DNA extracted from vaginal swabs. This yielded 11,350,333 reads across 202 samples (range 53 - 96,455; **Supplementary Table 1**). Following denoising and ASV assignment, we identified 727 features at a total frequency of 6,681,294 across 201 samples (median frequency – 33,790 per sample; full range - 27 to 66,127 per sample), of which 41 were deemed to be contaminant artifacts and subsequently removed. One negative control sample (ddH2O only control) was filtered from the dataset during denoising. After filtering remaining control samples (n=11) and two samples of unknown GBS status (ID 119) from the dataset, 706 features remained across 188 samples, at a total frequency of 4,708,210 (median frequency - 25,472.5 per sample).

**Figure 1:**
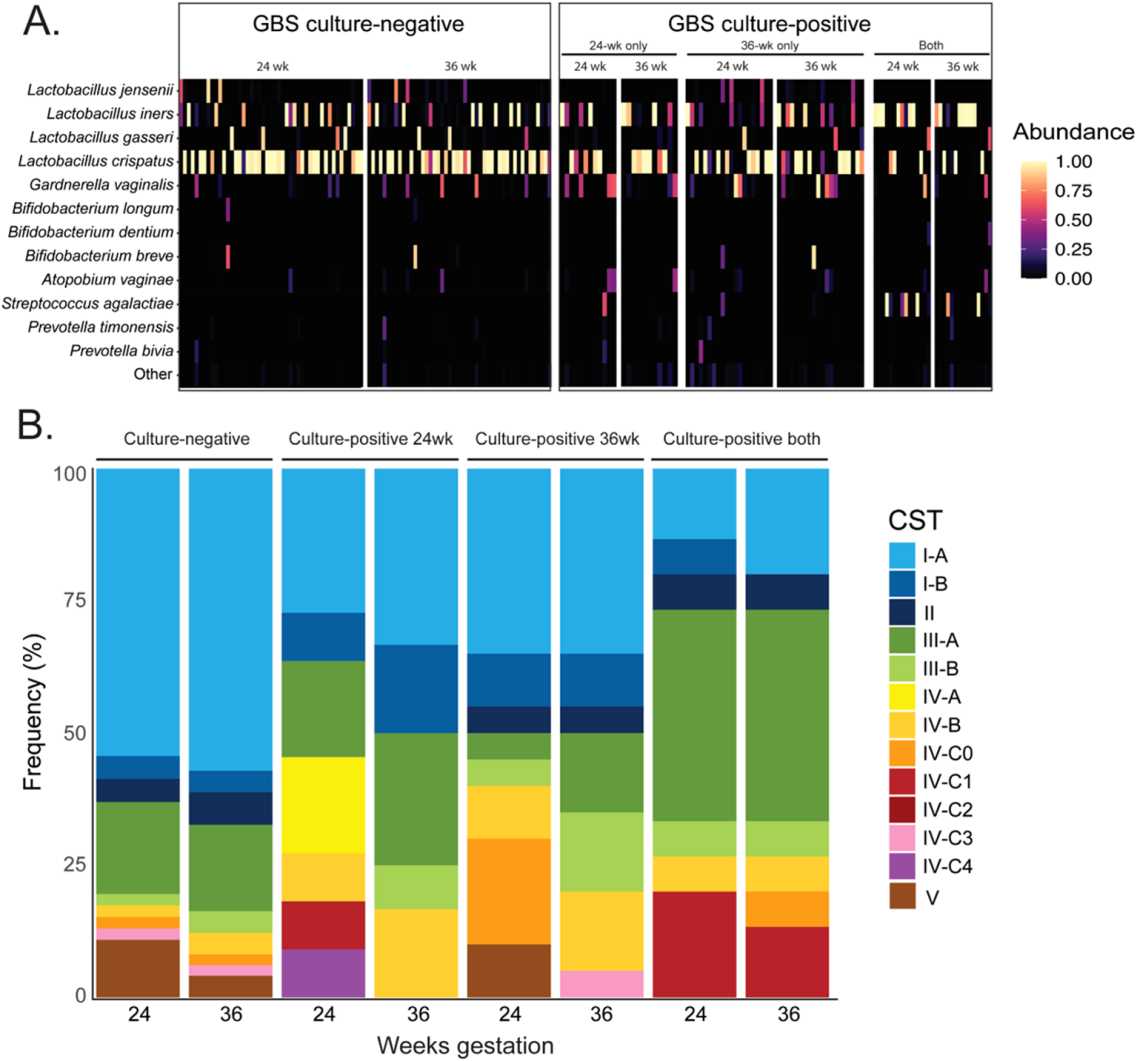
Relative abundance of bacterial species in the vagina of women GBS culture negative and culture positive throughout pregnancy and the distribution of vaginal community state types. **(A)** shows a heat-map representation of bacterial relative abundance in individual vaginal samples, grouped by GBS culture status and gestational timepoint. **(B)** displays the proportional composition of vaginal community state types within each group at 24- and 36- weeks’ gestation. CST – Community state type; GBS – Group-B streptococcus (*Streptococcus agalactiae*). CST I-A: Higher % *L. crispatus*; CST I-B: Lower % *L. crispatus*; CST II: *L. gasseri* dominant; CST III-A: Higher % *L. iners*; CST III-B: Lower % *L. iners*; CST IV-A: moderate *Gardnerella vaginalis* and BV-associated bacteria-1; CST IV-B: moderate *Atopobium vaginae* and *G*. *vaginalis*; CST IV-C0: Diverse with *Prevotella* spp. present; CST IV-C1: *Streptococcus* spp. present; CST IV-C2: *Enterococcus* spp. present; CST IV-C3: *Bifidobacterium* spp. dominant; CST IV-C4: *Staphylococcus* spp. present; CST V: *L. jensenii* dominant.

**Figure 1A** displays the relative abundance of the top 12 most abundant bacterial species. This highlights the high abundance of *L. crispatus* in the vaginal microbial community of GBS negative pregnant women but not in women who were persistently colonised with GBS at both collection timepoints.

To further characterise the vaginal microbial communities, we examined the community state types at both timepoints (CSTs; **Figure 1B**). Full taxonomic composition in each table can be found in **Supplementary Tables 2**–**5**. In the GBS negative group (n = 46), CST-I (*L. crispatus* dominant) represented the most prevalent CST (60%), followed by CST-III (*L. iners* dominant; 20%), CST-V (*L. jensenii* dominant; 7.37%), and CST-II (*L. gasseri* dominant; 5.26%). A non-*Lactobacillus* CST (-IV) was also identified in 7.4% of samples, with subtypes consisting of IV-B (*Gardnerella vaginalis* dominant; 1 sample at 24 weeks, 2 at 36 weeks), IV-C0 (even community with *Prevotella* spp.; 1 sample at each timepoint), and IV-C3 (*Bifidobacterium* dominant; 1 sample at each timepoint). Overall, the vaginal microbial community of GBS negative women remained stable between the 24- and 36-week timepoints, displaying no significant changes in community richness, and no significantly differentially abundant taxa (**Figure 1B**).

In the GBS positive 24-week group (n = 11), CST-I was the most prevalent vaginal CST at both gestational timepoints (43.5% total) followed by CST-III (26.1%), and CST-IV (30.4%; **Figure 1B**). Despite cultivation results, GBS was identified in just one individual sample at 24-weeks’ gestation (relative abundance – 59.2%) and one at 36-weeks (0.3% relative abundance). All samples containing *Streptococcus* spp. presented either CST-III or IV, with none exhibiting *L. crispatus* (CST-I) dominance. A decrease in the number of samples exhibiting CST-IV was observed at the 36-week timepoint in this group (GBS 24 wk group).

The GBS positive 36-week group contained 21 paired samples and CST-I represented the most common *Lactobacillus*-dominated vaginal CST in this group at both 24- and 36-weeks’ gestation (45% samples at both), followed by CST-III (24 weeks - 10%, 36 weeks – 30%), CST-V (24 weeks - 10%, 36 weeks – 0%), and CST-II (5% both; **Figure 1B**). A non-lactobacilli dominated CST-IV was identified in 30% and 25% of subjects at 24 weeks and 36 weeks’, respectively. These CST-IV samples were comprised of the subtypes IV-B (24 week – 10%, 36 week – 15%), IV-C0 (24 week – 20%, 36 week – 0%), and IV-C3 (24 week – 0%, 36 week – 5%). While there were no significant changes in community richness between 24- and 36-week samples in this group, the abundance of *G. vaginalis* was significantly more abundant at 24-weeks’ gestation compared to 36 weeks’ (Wald P-adj < 0.01). Interestingly, the 24-week timepoint samples in this group (GBS 36 wk), which were GBS culture negative, had a higher frequency of participants that had a diverse microbial community (CST-IV).

**Figure 2:**
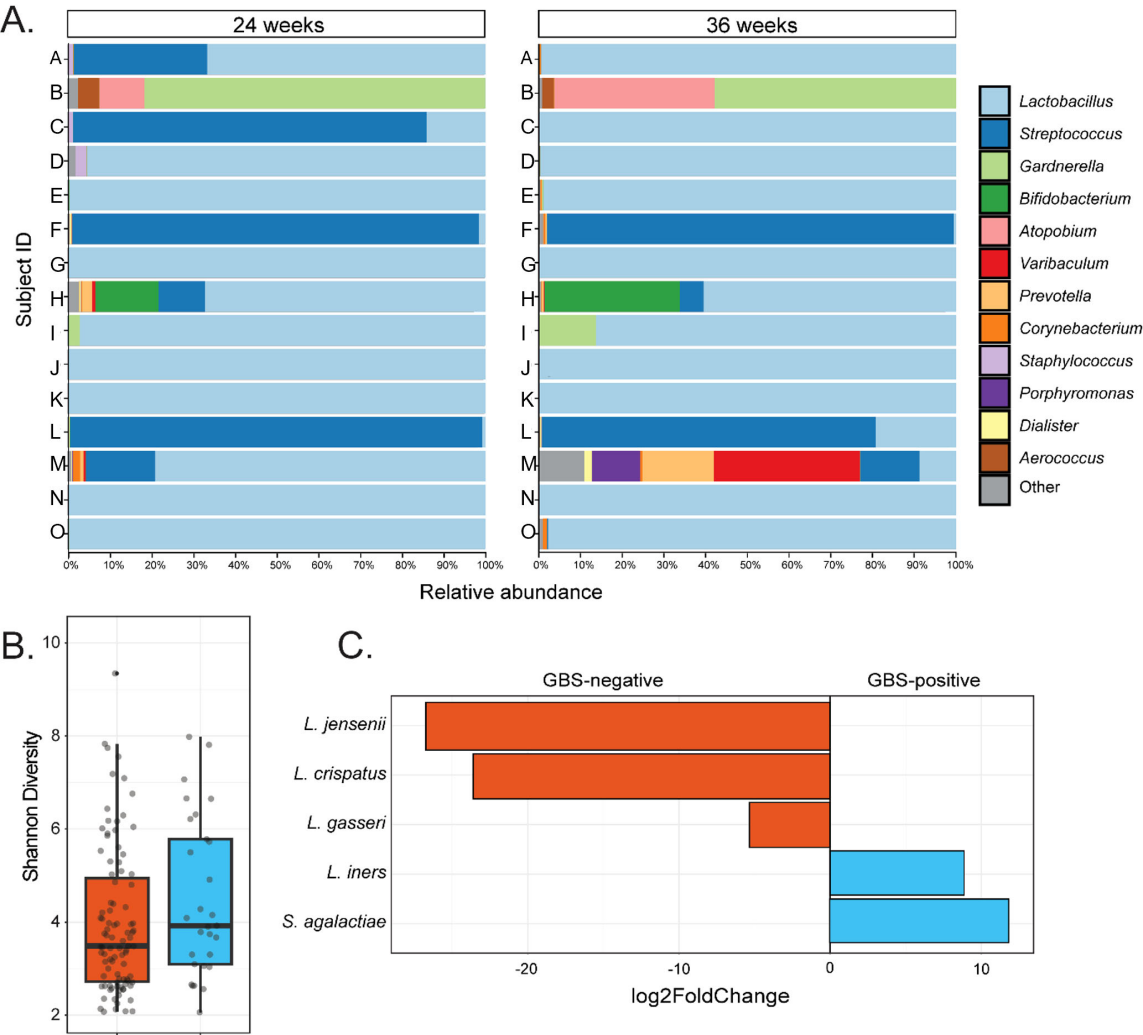
Taxonomic and compositional features of the vaginal microbiota in women GBS culture positive at both 24- and 36-weeks’ gestation. **(A)** Shows genus-level taxonomic bar plots for all individuals in the GBS 24/36 wk group, faceted by gestational timepoint. **(B)** Shows difference in Shannon diversity (alpha diversity) between GBS culture negative and the GBS positive at 24- and 36-week groups. **(C)** Displays taxa which significantly (Wald P-adj < 0.05) differed in abundance between GBS culture negative samples and the samples that were culture positive at both gestational timepoints, with degree of difference displayed on the X-axis as log2foldChange.

The most prevalent vaginal microbial CST in the GBS positive 24/36-week group (n = 15) was CST-III (*L. iners* dominated) which was identified in 46.7% of samples at both gestational timepoints (**Figure 1B**). CST-IV represented the next most prevalent compositional profile in this group with a prevalence of 26.7% at both timepoints, followed by CST-I (20% - both timepoints), and CST-II (6.7% - both timepoints). GBS was detected by 16S rRNA gene amplicon sequencing in the vaginal microbiota of 11/15 subjects in this group for at least one gestational timepoint, seven of which were positive at both, three at 24 weeks’ only, and one at 36 weeks’ only (**Figure 2A**; **Table 2**). As observed in other groups, vaginal microbial community richness remained stable between gestational time points, with only three individuals exhibiting shifts between CSTs (excluding changes within *Lactobacillus-*dominant CST subtypes).

**Table 2:** Relative abundances of *S. agalactiae* and serotype in the vaginal microbiota of culture-positive samples, ordered based on their corresponding community state type at 24- and 36-weeks gestation.

Subject ID	24 weeks gestation			36 weeks gestation		
	*S. agalactiae* relative abundance	CST	Serotype	*S. agalactiae* relative abundance	CST	Serotype
**N**	0.03%	I-A	V	0.00%	I-A	V
**G**	0.00%	I-A	NT	0.00%	I-A	NT
**M**	15.12%	I-B	Ia	12.00%	IV-C0	Ia
**H**	10.53%	II	Ia	5.59%	II	Ia
**K**	0.02%	III-A	Ia	0.00%	I-A	Ia
**J**	0.01%	III-A	IX	0.01%	III-A	IX
**E**	0.01%	III-A	ND	0.01%	III-A	ND
**O**	0.00%	III-A	ND	0.07%	III-A	ND
**I**	0.00%	III-A	NT	0.00%	III-B	NT
**D**	0.00%	III-A	VII	0.00%	III-A	ND
**A**	31.97%	III-B	V	0.00%	III-A	V
**B**	0.00%	IV-B	ND	0.00%	IV-B	ND
**L**	98.83%	IV-C1	III	79.72%	IV-C1	III
**F**	97.49%	IV-C1	III	97.46%	IV-C1	III
**C**	84.70%	IV-C1	V	0.02%	III-A	V

NT, non-typable capsular serotype. ND, Not done due to issues with sub-culturing from Brilliance GBS chromogenic agar.

### Higher abundance of *L. iners* and lower abundance of other *Lactobacillus* spp. is associated with persistent GBS colonisation

The relative abundance of GBS in samples that were culture-positive at both gestational timepoints varied greatly between individuals, ranging from 98.8% to 0.01% (median – 8.1%; **Figure 2A**). In subjects where GBS was detected via 16S rRNA sequencing in the vaginal microbiota in at least one timepoint, the median relative abundance of GBS was higher at 24 weeks’ gestation (median = 10.5%) than was observed at 36 weeks’ (median – 0.05%), with an average decrease in GBS relative abundance of 11% from 24 to 36 weeks’ gestation (**Table 2**). Interestingly, in those women persistently colonised with GBS, the same serotype was detected in 60% of women at both timepoints (20% serotype 1a (3/15); 13% serotype III (2/15); 20% serotype V (3/15); 7% serotype IX (1/15); 13% non-typable (i.e. GBS growth, but no serotype confirmed; 2/15)). There were a small number (20%; 3/15) of samples that were unable to be serotyped as they would not successfully subculture from the chromogenic agar and the colonies taken from the chromogenic agar would not react with the serotyping kit, possibly due to the proprietary chromogens in the agars affecting viability and capsular expression. One sample (7%) was only able to be serotyped at one of the timepoints.

To identify differences in the vaginal microbial communities associated with persistent GBS colonisation, we performed group comparisons between GBS positive at 24- and 36-week group and GBS negative group’s vaginal samples with respect to phylogenetic diversity, community structure, and differential abundance testing. We identified no significant differences in community richness (Shannon diversity; Kruskal-Wallis P > 0.05; **Figure 2B**) or structure (Unweighted UniFrac; ANOSIM P > 0.05) between GBS culture-negative and culture-positive groups at both single and grouped timepoints. However, differential abundance testing identified significantly higher abundances of *L. iners* (Wald P-adj < 0.05), and *S. agalactiae* (Wald P-adj < 0.001), as well as significantly lower abundances of *L. crispatus* (Wald P-adj < 0.001)*, L. gasseri* (Wald P-adj < 0.05), and *L. jensenii* (Wald P-adj < 0.001) in GBS positive samples compared to GBS negative samples (**Figure 2C**).

## Discussion

This study shows the dynamics of the vaginal microbial community during the second and third trimesters of pregnancy, in relation to GBS colonisation. The relationship between GBS and other bacterial taxa in the vagina is complex and poorly understood. Here we have demonstrated that the vaginal microbial community profile in women persistently colonised with GBS had a greater abundance of *L. iners*, compared to other *Lactobacillus* spp. in women not colonised with GBS. The vaginal microbial community tended to be more diverse in women exhibiting transient vaginal GBS colonisation, though not significant. The strength of our study is the longitudinal nature, in relation to GBS colonisation.

The composition of the vaginal microbial community changes throughout pregnancy, with increased abundance of *Lactobacillus* species and decreased abundance of anaerobic species (Romero et al., 2014). A longitudinal study from four timepoints during pregnancy demonstrated a pronounced shift in the CST composition with advancing gestational age, with those that were originally CST IV (diverse bacteria with no *Lactobacillus* spp.) becoming CST I (*L. crispatus* dominated) or CST III (*L. iners* dominated) by the end of the pregnancy (Romero et al., 2023). In line with previous studies, while we saw slight variations in the abundance of *L. crispatus* or *L. iners* between the two timepoints, each group of women had a relatively stable *L. crispatus/L. iners* dominance throughout pregnancy.

Vaginal metagenomic sequencing confirms GBS has a positive co-occurrence with *L. iners* and negative co-occurrence with *L. crispatus* (Pace et al., 2021). Culture of vaginal swabs has also shown GBS presence to be inversely related to any *Lactobacillus* species, with detection of *L. crispatus* being particularly uncommon in GBS positive samples (Starc et al., 2022). Accordingly, we observed that women persistently colonised with GBS had significantly decreased representation of *L. crispatus* compared to women with no or transient colonisation. Women who were GBS positive at 24 weeks only, tended to have more diverse vaginal microbial communities compared to GBS negative women, though not significant, and by 36 weeks, when GBS was not detected, the vaginal microbial community had shifted and was dominated by a combination of *L. crispatus* and *L. iners.* In contrast to our findings, a 16S rRNA vaginal microbiome study of pregnant women in Egypt showed that *L. iners* was predominant in GBS culture-negative women (Shabayek et al., 2022). However, GBS positive women did have a more diverse, less homogenous vaginal microbial communities, with a significant decrease in *Lactobacillus* spp. abundance and significantly higher *Ureaplasma*, *Gardnerella*, *Streptococcus*, *Corynebacterium*, *Staphylococcus*, and *Peptostreptococcus* genera (Rosen et al., 2017; Shabayek et al., 2022), similar to what we observed in women GBS positive at 24 weeks’ only. It is also well established that ethnicity and geographic location can influence vaginal microbiota composition, with differences in predominant CST commonly observed between regions (Roachford et al., 2022).

Reduced taxonomic diversity and a *Lactobacillus* spp. dominance in the vaginal niche are a possible mechanism for protection against GBS colonisation (Brokaw et al., 2021). Reduced lactic acid producing-*Lactobacillus* spp. dominance causes an increase in the vaginal pH (Ronnqvist et al., 2006; Ravel et al., 2011). *In vitro* studies have demonstrated that GBS binds to vaginal epithelial cells at a 4-fold higher rate when the pH is elevated (Park et al., 2012). Additionally, fluctuations in pH, from acidic to neutral, cause an upregulation of expression of numerous GBS virulence factors, which in turn can change GBS from an asymptomatic carriage state to a virulent invasive state (Brokaw et al., 2021).

*L. iners* is commonly detected in specimens from women with BV and is often considered a transitional species that provides less protection against pathogens than other *Lactobacillus* spp (Ferris et al., 2007; Zozaya-Hinchliffe et al., 2010).. For example, *L. iners* is only able to produce L-lactic acid, compared to *L. crispatus, L. gasseri* and *L. jensenii* which can make both L-lactic acid and D-lactic acid (France et al., 2016). The production of D-lactic acid is suggested to have greater inhibitory effect on exogenous bacteria than L-lactic acid (Zheng et al., 2021). *L. crispatus* can also generate antibacterial hydrogen peroxide, where *L. iners* cannot, again highlighting that *L. iners* is not as effective at protecting the vaginal environment against opportunistic pathogens such as GBS (France et al., 2016; Zheng et al., 2021). In cases of BV, *L. iners* often coexists with other potentially harmful bacteria associated with poor pregnancy outcomes and is not easily displaced by pathogens (Ferris et al., 2007; Zozaya-Hinchliffe et al., 2010). Co-infection with *G. vaginalis*, the most abundant member of a dysbiotic microbiota, and GBS, led to a 10-fold higher risk of GBS vaginal colonisation in a pregnant mouse model, with 40% of co-infected mice exhibiting ascending GBS infection of the uterus and placenta (Gilbert et al., 2021). In our study, women who were GBS positive at any or all timepoints examined displayed an increase in placental abnormalities detected. GBS infection of the uterus and placenta are associated with poor pregnancy outcomes including premature rupture of membranes, pre-term birth, clinical chorioamnionitis and neonatal infection (Bianchi-Jassir et al., 2017; Brokaw et al., 2021; Mei and Silverman, 2023). This highlights the importance of understanding how GBS can colonise a unique environmental niche, such as the vagina, and subsequently ascend to cause such sequalae.

In women that were persistently colonised with GBS there was an average decrease of 11% of GBS abundance as pregnancy progressed. It is established that the diversity of the vaginal microbial community decreases as pregnancy progresses (Romero et al., 2014; Romero et al., 2023). It has been suggested that this is hormonally driven, with increasing lactobacilli abundance associated with an increase in oestrogen (Prince et al., 2015). However, this is a field that is also lacking and requires far more detailed research to determine what drives the decrease in microbial diversity or increases in lactobacilli abundance as pregnancy progresses.

Our study has several strengths and limitations that need to be acknowledged. The cohort that the samples used here were collected from were a group of mostly healthy women, where extensive data such as patient demographics, medical usage and neonatal outcomes were collected (Borg et al., 2021). Importantly, these samples were able to be collected in a longitudinal manner, allowing us to examine changes in the vaginal microbial communities throughout pregnancy, in relation to GBS colonisation. In the state of Queensland, Australia, GBS screening is not recommended as part of the standard perinatal care, and a risk-based approach is used instead (Guidelines QC, 2022). This means that there are no official diagnostic records for GBS colonisation status. We did however use the diagnostic standard agar culture to differentiate our groups. Furthermore, we completed PCRs on the samples targeting the highly conserved *sip* gene (data not shown), confirming our culture results. As with any study involving human participants, larger sample sizes are always preferred. We included all eligible patient samples in this study and have examined the vaginal microbial community in relation to GBS colonisation, longitudinally throughout pregnancy. There are numerous V3-V4 primer sets that have been used for vaginal microbiome determination (Graspeuntner et al., 2018; Van Der Pol et al., 2019; Hugerth et al., 2020), with little consistency between studies. The primers used here were chosen based on broad coverage of diverse vaginal taxa (Graspeuntner et al., 2018; Van Der Pol et al., 2019). The primers that we used were unfortunately not highly specific towards clinical GBS isolates, which meant that for some samples where GBS was cultured, the sequencing was not able to specifically identify all isolates of GBS (**Table 2**). The primers were originally selected because of their ability to identify a wide range of bacterial species in the vaginal niche when compared to the use of V1-V2 primers (Graspeuntner et al., 2018).

## Conclusions

Very few studies have examined vaginal microbial communities in relation to GBS colonisation during pregnancy (Shabayek et al., 2022), and this has only been done at a single timepoint during pregnancy, usually in the last trimester of pregnancy, preventing temporal changes from being detected (Shabayek et al., 2022; McCoy et al., 2023). The relationship between GBS and other bacterial taxa in the vagina is complex and poorly understood. Here, we have shown for the first time how the vaginal microbial communities change throughout pregnancy with changes in GBS colonisation status, and that GBS colonisation may be associated with a reduction in *L. crispatus, L. gasseri* and *L. jensenii* dominance in comparison to women who were not colonised with GBS at either timepoints. In women that were persistently colonised with GBS throughout pregnancy, we demonstrated a significant increase in *L. iners* and significant reduction in *L. crispatus* abundance. Given the lack of understanding of how the vaginal microbial community contributes to or prevents GBS colonisation, it is imperative to further investigate how *L. iners* and GBS interact in this unique environmental niche.

## Data Availability

The datasets presented in this article are not readily available as the participants of this study did not give written consent for their clinical data to be shared publicly, so due to the sensitive nature of the research, supporting data is not publicly available. Access to raw sequencing reads and metadata may be achieved upon request to the corresponding author and consultation with the QFC governance committee.
